# Exploration of the Yield Potential of Mesoamerican Wild Common Beans From Contrasting Eco-Geographic Regions by Nested Recombinant Inbred Populations

**DOI:** 10.3389/fpls.2020.00346

**Published:** 2020-04-03

**Authors:** Jorge Carlos Berny Mier y Teran, Enéas R. Konzen, Antonia Palkovic, Siu M. Tsai, Paul Gepts

**Affiliations:** ^1^Department of Plant Sciences, University of California, Davis, Davis, CA, United States; ^2^Cell and Molecular Biology Laboratory, Centro de Energia Nuclear na Agricultura, Universidade de São Paulo, Piracicaba, Brazil

**Keywords:** common bean, crop wild relative, eco-geographic adaptation, nested backcrossed inbred populations, quantitative trait loci, yield

## Abstract

Genetic analyses and utilization of wild genetic variation for crop improvement in common bean (*Phaseolus vulgaris* L.) have been hampered by yield evaluation difficulties, identification of advantageous variation, and linkage drag. The lack of adaptation to cultivation conditions and the existence of highly structured populations make association mapping of diversity panels not optimal. Joint linkage mapping of nested populations avoids the later constraint, while populations crossed with a common domesticated parent allow the evaluation of wild variation within a more adapted background. Three domesticated by wild backcrossed-inbred-line populations (BC_1_S_4_) were developed using three wild accessions representing the full range of rainfall of the Mesoamerican wild bean distribution crossed to the elite drought tolerant domesticated parent SEA 5. These populations were evaluated under field conditions in three environments, two fully irrigated trials in two seasons and a simulated terminal drought in the second season. The goal was to test if these populations responded differently to drought stress and contained progenies with higher yield than SEA 5, not only under drought but also under water-watered conditions. Results revealed that the two populations derived from wild parents of the lower rainfall regions produced lines with higher yield compared to the domesticated parent in the three environments, i.e., both in the drought-stressed environment and in the well-watered treatments. Several progeny lines produced yields, which on average over the three environments were 20% higher than the SEA 5 yield. Twenty QTLs for yield were identified in 13 unique regions on eight of the 11 chromosomes of common bean. Five of these regions showed at least one wild allele that increased yield over the domesticated parent. The variation explained by these QTLs ranged from 0.6 to 5.4% of the total variation and the additive effects ranged from −164 to 277 kg ha^–1^, with evidence suggesting allelic series for some QTLs. Our results underscore the potential of wild variation, especially from drought-stressed regions, for bean crop improvement as well the identification of regions for efficient marker-assisted introgression.

## Introduction

Among pulses, common bean (*Phaseolus vulgaris* L.; 2*n* = 2*x* = 22) plays an important nutritional and economical role (Broughton et al., 2003; Gepts et al., 2008). The yields of pulses are usually lower than those of cereals, mainly because their production is located in more marginal cultivation niches, produce more energy-dense seeds and the cost of association with nitrogen fixing rhizobia (Sinclair and Vadez, 2012). Production is also constrained by biotic and abiotic factors, drought being one of the main causes of yield reduction and crop failure in beans (Singh, 2001; Beebe et al., 2013; Ramirez-Cabral et al., 2016). Furthermore, drought severity is likely to increase due to the effects of climate change (Prudhomme et al., 2014). Increasing crop yield and resilience is an essential goal of crop breeding and cultivar development, as well as a direct advantage to farmers and, ultimately, to consumers (Kissoudis et al., 2016). Several strategies to improve yield include maximizing nitrogen fixation, photosynthesis and partitioning to grain, as well as minimizing water deficit impacts (Monteith and Moss, 2006; Zhu et al., 2010; Sinclair and Vadez, 2012).

Common bean is one of the five domesticated species among ∼80 wild *Phaseolus* species, all originating in the American continent (Freytag and Debouck, 2002; Delgado-Salinas et al., 2006). Wild common bean originated in Mexico and was dispersed by at least two long-distance movements to Ecuador and northern Peru (some 500,000 years ago) and the southern Andes (southern Peru, Bolivia, and northwestern Argentina; some 100,000 years ago) leading to two additional gene pools in the Andes (Bitocchi et al., 2012; Rendón-Anaya et al., 2017; Ariani et al., 2018; Gepts, 2019). Common bean was domesticated independently within two of the three gene pools (Southern Andes and Mesoamerica), followed by divergence into six genetically distinct races (Kwak and Gepts, 2009). Mesoamerican beans were presumably domesticated in Western Mexico while Andean beans were domesticated in northern Argentina and southern Bolivia (Kwak et al., 2009; Rodriguez et al., 2016). Within the domesticated Mesoamerican gene pool, the larger-seeded races ‘Jalisco’ and ‘Durango’ are distributed in the sub-humid and semi-arid highlands, respectively, in Central and Northern Mexico, while the small-seeded race Mesoamerica is distributed in the lowlands from southern Mexico to northeast Brazil (Singh et al., 1991). Within each race, there are thousands of landrace types, as well as fewer, but established, commercial market classes (Gentry, 1969; Singh et al., 1991; Moghaddam et al., 2016). In common bean breeding, the variation included in the breeding programs is mostly constrained within market classes and within races, as the inheritance of color, size, and shape of seeds and plant architecture are highly polygenic and dispersed throughout the genome (Park et al., 2000; McClean et al., 2002; Checa and Blair, 2008; Schmutz et al., 2014). Introgressions between market classes or gene pools have focused mostly on transferring disease resistance, modifying growth habit, and introducing drought resistance (Kelly, 2001; Acosta-Gallegos et al., 2007; Beebe, 2012; Porch et al., 2013; Dohle et al., 2019).

The use of wild variation has been even more limited. Wild variation has been identified as a source of resistance to bruchids (Kornegay et al., 1993; Osborn et al., 2003, 2006), common bacterial blight (Beaver et al., 2012), web blight (Beaver et al., 2012) and white mold (Mkwaila et al., 2011). However, it is possible that beneficial wild variation for highly quantitative important traits, like grain yield and drought adaptation, is not present the domesticated forms due to early genetic bottlenecks (Gepts et al., 1986; Tanksley and McCouch, 1997; Acosta-Gallegos et al., 2007). For example, within the Mesoamerican gene pool, a single domestication event originated in only one of the three genetically and geographically distinct groups of wild common beans (Kwak et al., 2009; Ariani et al., 2018). Although domesticated forms expanded to areas of those non-domesticated, wild groups, and can outcross with the wild relatives, gene flow has been found highly asymmetrical, introgressing more regions from the domesticated to the wild types (Papa and Gepts, 2003). Therefore, diversity unique to the non-domesticated groups might not be present in the domesticates. This is supported by a strong genetic diversity bottleneck, especially during the Mesoamerican bean domestication (Gepts et al., 1986; Sonnante et al., 1994; Schmutz et al., 2014). Previous efforts in genetic analyses in common bean using wild by domesticated crosses include: (a) two populations with the same Andean domesticated cultivar (ICA Cerinza) crossed to a wild type from Colombia (Blair et al., 2006) and to one from northern Mexico (Blair and Izquierdo, 2012); (b) a population resulting from a cross of Midas (an Andean snap bean), and a wild type from central Mexico G12873 (Koinange et al., 1996); and (c) a population involving Peruvian accessions both originating in the Andean gene pool (Singh et al., 2019). In the first two efforts, genomic regions carrying a positive-effect allele for yield from the wild parent were detected in the wild accession from a high rainfall area in Colombia (Blair et al., 2006), but not in the accession from a low rainfall region in northern Mexico (Blair and Izquierdo, 2012).

In the present investigation, the development of three backcrossed-inbred-line populations in a nested design is described, as is their evaluation under drought pressure in field conditions followed by a QTL analysis of grain yield. The populations result from the cross between three wild Mesoamerican accessions originating from areas with different levels of precipitation/evapotranspiration to the same elite breeding line, also of Mesoamerican origin. The nested design allows the sampling of more diversity than single biparental populations, increases the power and mapping resolution, but most importantly allows the testing of genetic effects among accessions in similar genetic backgrounds (Yu et al., 2008; Garin et al., 2017). Our two-fold hypothesis is that: (1) The three wild accessions have different adaptations to rainfall/evapotranspiration regimes; and (2) Some of these contrasting adaptation mechanisms were not included into the domesticated gene pool during domestication.

## Materials and Methods

### Parental Materials and Population Development

Three populations were established, which resulted from a cross of the Mesoamerican elite breeding line SEA 5 with three wild accessions from the Mesoamerican gene pool. SEA 5 (PI 613166; Number and DOI of the CIAT gene bank: G51502, doi: 10.18730/PHA81) was obtained at the International Center for Tropical Agriculture (CIAT, Cali, Colombia) from an interracial (Durango × Mesoamerica) double cross (BAT 477/San Cristobal 83//Guanajuato 31/Rio Tibagi) within the Mesoamerican gene pool (Singh et al., 2001). SEA 5 was selected for high productivity under drought, has an indeterminate inclined growth habit IIb, photoperiod neutrality, resistance to Fusarium root rot and Bean Common Mosaic Virus (*I* gene), but shows susceptibility to anthracnose, common bacterial blight and rust (Singh et al., 2001; Terán and Singh, 2002). SEA 5 develops a vigorous and deep root system (Polania et al., 2017), has a high capacity of photoassimilate remobilization (Polania et al., 2016; Rao et al., 2017), and a high capacity for nitrogen fixation under drought (Devi et al., 2012).

Three wild accessions within the Mesoamerican wild gene pool (Ariani et al., 2018; Berny Mier y Teran et al., 2019a) were chosen to maximize genetic variation and annual precipitation of their collection site. From North to South and along a gradient of increasing temperature and rainfall, the wild accessions included PI 319441 (G10022, DOI: 10.18730/JRH59), collected in the state of Durango, Mexico (104.47°N, 24.24°W, mean annual temperature: 18°C, mean annual precipitation: 588 mm), PI 417653 (G12910, DOI: 10.18730/PGEK4) from Guanajuato, Mexico (101.72°W, 20.62°N, 19.4°C, 733 mm) and PI 343950 from Huehuetenango, Guatemala (91.82°N, 15.68°W, 23.5°C, 1600 mm). The seeds were obtained from the National Plant Germplasm System (NPGS) of the USDA at the Western Regional Plant Introduction Station in Pullman, WA, United States. Climatic conditions of each collection site were extracted from the WorldClim database^1^ (Hijmans et al., 2005).

The three populations (henceforth called dw319441, dw417653, and dw343950) were developed in an identical way as follows: (1) SEA 5 was crossed to the wild accession using SEA 5 as the male parent; (2) The F_1_ plant was then used as the male parent crossed back to SEA 5 plants to obtain at least 250 BC_1_ seeds; (3) Each BC_1_ seed was scarified before planting and allowed to self in the greenhouse; (4) BC_1_S_1__:__2_ families were grown in the field in the summer months (June–September, short night/long day photoperiod) planting ∼50 seeds per family, to allow selection against photoperiod sensitivity. Seeds from a randomly chosen plant from each family were harvested; (5) The plants were grown in the greenhouse for two more cycles using single-seed-descent to obtain 220, 237, and 238 BC_1_S_4_ plants from the dw319441, dw417653, and dw343950 populations, respectively.

### Trial Design and Phenotyping

The populations were evaluated under field conditions in 2014 and 2015 at the Plant Sciences Field Facility on the University of California, Davis, campus (38.53°N, 121.78°W). The soil type of the site belongs to the Yolo series, a member of fine-silty loam, mixed, non-acid, thermic Mollic Xerofluvents, considered well-drained, with slow to medium runoff and moderate permeability^2^. The seeding was carried out on the 8th of June in 2014 and on the 9th of June in 2015. The plants were harvested on the 22–26th of September in 2014 and the 14–25th of September in 2015. In 2014, the populations were evaluated only under full irrigation, using furrow water delivery as needed, in four irrigations. In 2015, a terminal drought water regime was added. Terminal drought was simulated by withdrawing irrigation in the last two of the four irrigations of the full irrigated treatment. Plants received water only from irrigations as there were no rain events during the experiments. The agricultural management was according to standard practices in California (Long et al., 2010).

In both years, the RILs were planted in un-replicated fashion and SEA 5 in replicated fashion (eight replicates) according to a modified augmented design (Lin and Poushinsky, 1985). The field was divided in blocks and planted with a main check in the middle of each block, as well as two to three secondary checks randomly distributed within each block. In 2014, 30 blocks of 3 plots × 9 plots were used and, in 2015, 21 blocks of 5 plots × 5 plots per treatment were used. UCD 9634, a pink-seeded breeding line, was included in both years as the main check due its high yield and stability. In 2014, Tio Canela 75 (Rosas et al., 2010), Matterhorn (Kelly et al., 1999), and Flor de Mayo Eugenia (Acosta Gallegos et al., 2010) were included as secondary checks. In 2015, a small, black-seeded line L88-63 (Frahm et al., 2004) was added as a check. The experimental unit was a plot of 60 plants grown in a row of 6 m-long rows and 0.76 m between rows (density of 131,578 plants per hectare). In 2014, 230, 238, and 237 progeny lines of the dw319441, dw417653, and dw343950 populations, respectively, were grown.

**FIGURE 1:**
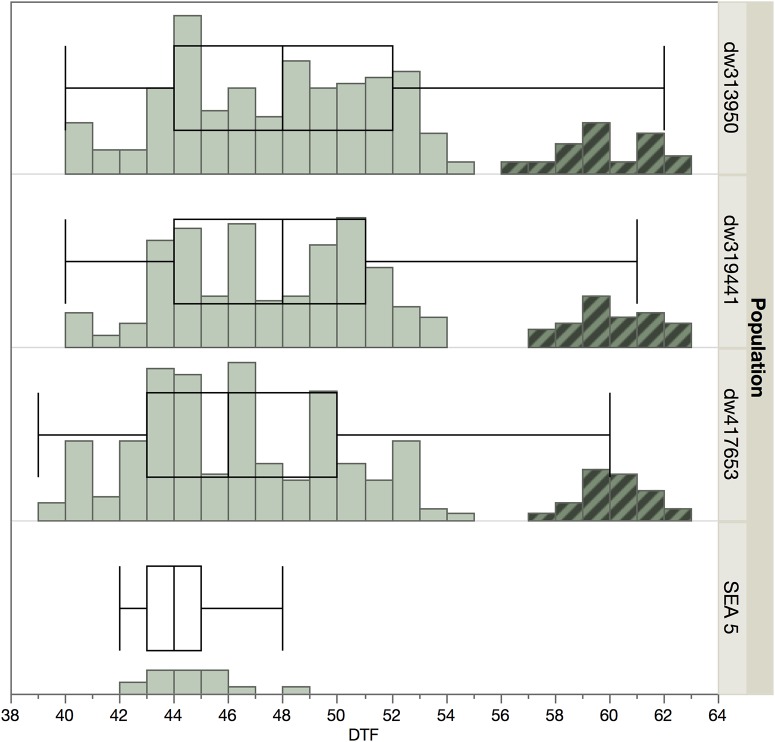
Distribution of flowering time among populations and SEA 5 in 2014. The highlighted accessions in the later part of the distribution were not planted in 2015 to minimize the effect of phenology.

There was a bimodal distribution in days to flowering (Figure 1) with a smaller set of lines that showed late flowering. These later genotypes were not used for further evaluation and genotyping, as a wide variation in phenology can be a confounding effect (Pinto et al., 2010), especially in the case of late flowering and maturity, which are negatively correlated with yield in beans (Klaedtke et al., 2012; Berny Mier y Teran et al., 2019b). The final analyses used 171, 170, and 165 lines from the dw319441, dw417653, and dw343950 populations, respectively.

Grain yield was measured per experimental plot and extrapolated to kilograms per hectare. Flowering time was taken when at least 50% of the plot had an open flower. Seed weight was evaluated in random subsample of 100 seeds.

### Statistical Analyses

To adjust for within-field spatial variation, a two-dimensional tensor product was used, i.e., the penalized splines (P-splines) method with the package SpATS (Rodríguez-Álvarez et al., 2018) in R (R Development Core Team, 2018). Briefly, the method fits the mixed model *y* = *X*β+*X*_*s*_β_*s*_+*Z*_*s*_*s*+*Z*_*u*_*u*+*Z*_*g*_*g*+*e*, where *β* and β*_*S*_* are vectors that include the intercept and check cultivar effect as fixed term, *X*_*S*_is the design matrix, *X*_*s*_β_*s*_ and *Z*_*s*_*s* are fixed and random components of the mixed model, respectively, *s* is the vector of random spatial effects, *u* is a random row and column effect sub-vector accounting for discontinuous variation, and *g* is the random genotypic effect (Malosetti et al., 2017; Rodríguez-Álvarez et al., 2018). The genotype was considered as a random effect in both years and irrigation treatment as a fixed effect in the second year. An analysis of variance was carried out as a linear mixed model using population, environment, and their interaction as fixed effects, and genotype within population as random effects. The evaluations in 2014 under full irrigation, in 2015 under terminal drought and full irrigation were considered as separate environments. The statistical analyses were performed with JMP (SAS Institute Inc., 2016). The Drought Susceptibility Index (DSI) calculated from the two irrigation treatments in 2015 as (1 – *Y*_*ds*_/*Y*_*ns*_)/1 – (*X*_*ds*_/*X*_*ns*_), where *Y*_*ds*_ and *Y*_*ns*_ are yields in drought stress and no stress environments, respectively, and *X*_*ds*_ and *X*_*ns*_ are the overall yield under drought stress and no stress treatments, respectively (Fischer and Maurer, 1978; Beebe et al., 2013).

### Genotyping

The BC-RILs, parents, and F_1_ hybrids were genotyped with the BARCBean6K_3 BeadChip platform of 5,398 SNP markers (Song et al., 2015) at the Genome Center at the University of California-Davis. After filtering in GenomeStudio Module v1.8.4 (Illumina Inc., San Diego, CA, United States), SNP calling was performed with the software’s cluster algorithm, with subsequent manual adjustments and a quality control with a 0.15 Gencall score cutoff. The markers were filtered for less than 10% missing data and polymorphism between parents, verified with the F_1_ hybrids.

### Map Construction

Linkage maps were constructed for each population in R (R Development Core Team, 2018) using the package asMAP (Taylor and Butler, 2017). Genetic distances were determined using the Kosambi function (Kosambi, 1944). The recombination fraction was estimated with qtl (Wu et al., 2003). A consensus map, combining results for the three individual maps, was developed with LPmerge (Endelman and Plomion, 2014), with the linkage maps of the three populations constructed without first filtering for co-located markers, to maximize the markers shared among populations. LPMerge uses linear modeling to keep the maximum interval with the lowest root mean squared error applying weights to population size (Endelman and Plomion, 2014). When there were co-localized markers in the consensus map, only one marker per bin was kept. Chromosomes were numbered Pv01 to Pv11 matching the standard numbering in *P. vulgaris* (Pedrosa-Harand et al., 2008).

### QTL Mapping

The QTL analysis was performed with the NAM function of QTL IciMapping version 4.1 (Meng et al., 2015). The NAM module implements joint inclusive composite interval mapping (JICIM), uses generalized linear models with population and marker by population interaction as fixed effects through stepwise regression for marker selection and subsequent interval mapping with adjusted phenotypes from the selected markers outside the current marker interval (Li et al., 2007, 2011; Wang et al., 2016). A probability of 0.01 and a step of 1 cM was used for the stepwise regression and the significance. The LOD threshold was calculated by 2,000 permutations in each environment at a significance level of 0.05. Furthermore, a QTL analysis was also conducted for the average yield across the three environments in 2014 and 2015 and the drought susceptibility index (DSI).

### Data Availability

The marker segregation and phenotypic evaluation data have been deposited in the Dryad public database: https://datadryad.org/stash/dataset/doi:10.25338/B8FW3M.

## Results

### Sources of Variation

The effects of population, environment, and their interaction were highly significant (Table 1). Nevertheless, the environmental effect was larger (*F* = 1,672, *P* < 0.001) than both population and their interaction (*F* = 5.7 and 3.6, at *P* < 0.01, respectively). Across environments, the yield of the dw417653 population was the highest, being significantly higher (113 kg, +11%) than the yield of the dw343950 population, whereas the dw319441 population was not statistically different from other two populations. Across populations, the yield in 2014 was significantly lower than both water regimes in 2015, with a reduction of 56 and 47%, relative to drought and well-watered treatments, respectively. There was a significant yield reduction in 2015 because of terminal drought, with 17% lower yield relative to the irrigated treatment.

**TABLE 1:** Descriptive statistics and analysis of variance for yield (kg ha^–1^) for three backcrossed inbred lines and the recurrent parent evaluated in three environments.

RIL	Mean	1082.3
	Min	469.1
	Max	1987.2
SEA 5	Mean	1575.7
*F*-values	Population	5.7**^1^
	Environment	1672.5***
	PxE	3.6**
Population mean	dw319441	1084.1 AB
	dw417653	1137.1 A
	dw343950	1024.1 B
Environmental means	2014C^3^	624.5 C^2^
	2015C	1434.0 A
	2015D	1186.7 B

^1^Significance codes: ***<0.001, **< 0.01. ^2^Levels not connected with the same letter are significantly different according to the Tukey-Kramer HSD test (*P* < 0.05). ^3^Treatments: full irrigation (C), terminal drought (D).

### Comparison of Populations Across Environments

**FIGURE 2:**
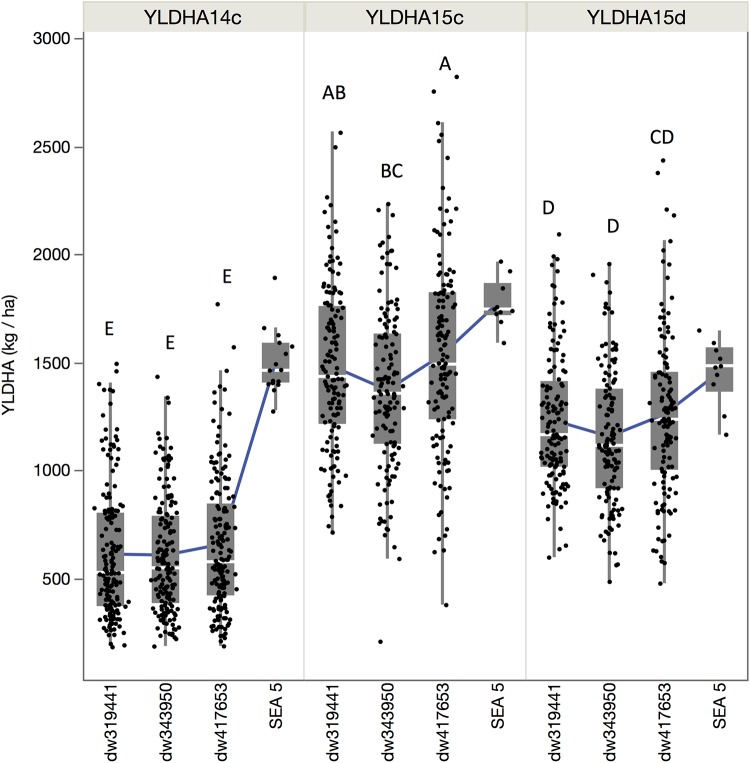
Quantile boxplot and overall mean (green line) of the yield of the three populations and recurrent parent for the three environments: fully irrigated in 2014 (YLDHA14c), fully irrigated in 2015 (YLDHA15c), and terminal drought in 2015 (YLDHA15c). Averages not connected with the same letter are significantly different according to the Tukey-Kramer HSD test (*P* < 0.05).

Although the population by environment effect was significant (Table 1), the ranks of the performance of the populations were similar between environments. Across environments, the dw417653 population produced higher yield than the dw319441 population, which, in turn, was higher than the dw343950 population. However, population dw417653 was significantly different from population dw343950 only in 2015 (Figure 2). In all environments, the average of the population yields was lower than that of SEA 5. Across environments, SEA 5 produced 45% more yield than the average of the RILs (Table 1).

For breeding purposes, some RILs showed transgressive segregation for higher yield compared to the average of SEA 5 plots in 2015, although none were higher than the highest yielding SEA 5 plot in 2014 (Figure 2). However, in 2015 in both treatments, some RILs yielded more than the highest plot of SEA 5. Furthermore, there were high-performing RILs from each population in both years and treatments (Figure 2). Two populations, dw417653 and dw319441, included progeny lines with yields that were significantly higher (+20% on average) – across the three environments – than that of the domesticated control SEA 5 (Table 2). These lines, nevertheless, had similar number of days to flowering (42 to 54 days vs. 47 for SEA 5) and only slightly smaller seeds than SEA 5) (15–20 g/100 seeds vs. 24 g/100 seeds for SEA 5; Table 2).

**TABLE 2:** Progeny, backcross-inbred lines with highest yields (kg ha^–1^) across the three environments [fully irrigated in 2014 (YLDHA14), fully irrigated in 2015 (YLDHA15c), and terminal drought in 2015 (YLDHA15d)].

**Population**	**Lines**	**100SW^1^**	**DTF^2^**	**Average over the three environments**		**2014 (well-watered)**		**2015 (well-watered)**		**2015 (drought-stressed)**	
				**YLDHAave**	**Rank**	**YLDHA14**	**Rank**	**YLDHA15c**	**Rank**	**YLDHA15d**	**Rank**
dw417653	p53-36	20	46	**1987**	1	1286	5	**2824**	1	**2379**	2
	p53-235	20	42	**1889**	2	**1572**	1	**2610**	3	**1956**	5
	p53-114	20	43	**1866**	3	1463	3	**2527**	5	**2063**	3
dw319441	p41-262	15	48	**1856**	4	1461	4	**2566**	4	**1993**	4
dw417653	p53-65	18	54	**1856**	5	824	6	**2756**	2	**2437**	1
Control	SEA 5	24	47	1576	6	1500	2	1773	6	1455	6

Difference between best progeny lines and SEA 5 yields				+20%		−12%		+50%		+49%	

*Yield values in bold represent yields that are significantly higher than those of the control. ^1^100SW, 100-seed weight (g). ^2^DTF, number of days to flowering.*

### Correlations Among Traits and Distribution

**FIGURE 3:**
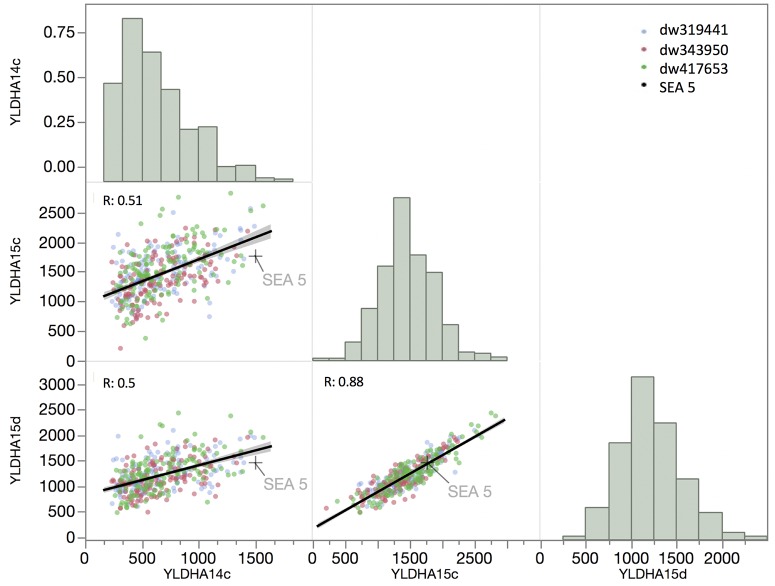
Matrix of trait frequency distribution (diagonal), joint distribution and correlation coefficient (lower triangle) of yield evaluated in 2014 under full irrigation (YLDAH14c) and in 2015 under full irrigation and terminal drought (YLDAH15c and YLDAH15d, respectively). Traits. The mean of SEA 5 is showed as plus (+) sign.

The correlation of yield between the 2014 and 2015 well-watered treatments and the 2015 terminal drought treatment was relatively low, with an R of 0.51 and 0.5, respectively (Figure 3), but the correlation between drought and well-watered conditions in 2015 was higher (*R* = 0.88). The overall distributions of yield among the RILs in the first year and in 2015 under drought were not normally distributed (*P* < 0.001, Shapiro–Wilks test) but were skewed toward the low- yielding side of the distribution, while the distribution of yield in well-watered plots in 2015 was normally distributed (*P* = 0.17).

### Molecular Linkage Map and QTL Analyses

#### Polymorphism, Recombination Rate and Allele Frequency

**FIGURE 4:**
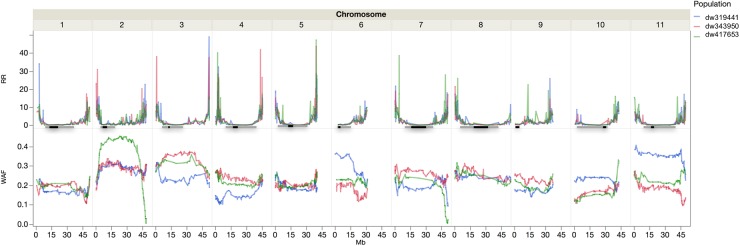
Recombination rate (RR, cM Mb^–1^) and wild allele frequency (WAF) of the three wild by domesticated populations. The horizontal lines represent the approximate location of the pericentromere (gray) and centromere (black) according to Schmutz et al. (2014).

There were 1554, 1404, and 1499 polymorphic markers between SEA 5 and PI 343950, PI 417563, and PI 419441, respectively. Jointly, there were 2,201 polymorphic markers between the wild accessions and SEA 5 and 1,858 markers shared among the three wild accessions. The trends in recombination rate were similar across populations. Higher recombination rates were observed in the distal parts of all the chromosomes, except on chromosome Pv06 where higher recombination was observed from the middle to the distal portion and chromosome Pv09 where higher recombination was located in the second and fourth quarter of the chromosome (Figure 4).

The wild allele frequency was variable between and within chromosomes. After one backcross to the domesticated parent, one would expect an average frequency of 0.25 of the wild parent. The average frequency was 0.23 across chromosomes, with a range of 0.17 to 0.4, in Pv01 and Pv02, respectively. The populations had similar frequencies across chromosomes; however, within chromosomes there were differences among populations. For example, in Pv02, compared to the other two populations, the dw417653 population showed higher wild allele frequency in the central region but very low frequencies at the chromosomal ends. In Pv10, the P319441 population had higher wild allele frequency than the other populations. In some chromosome regions, all three populations showed a lower than expected wild allele frequency, like in the distal region of Pv01. There were two regions with markedly low wild allele frequency, the region mentioned before in Pv02 and another region in Pv07, both at the end of the chromosomes.

#### Consensus Molecular Linkage Map

The consensus map was built with 721 markers and had a genetic length of 925 cM (Table 3). Per chromosome, there were on average 66 markers, an average length of 84 cM, and an average spacing of 1.3 cM. The average maximum spacing was 9 cM, while the largest interval was 14 cM in Pv01. Through a comparison of the range of the markers on each chromosome and their physical position according to the G19833 reference genome version 2.1 (Schmutz et al., 2014), the genetic map spanned 510,318,067 bp, which represents 99% of the sequenced genome. The coverage ranged from 96.5% of Pv06 to 99.7% of Pv08.

**TABLE 3:** SNP distribution among the linkage groups/chromosomes of the consensus map.

**Chromo-some**	**Number of markers**	**Length of linkage groups**	**Average spacing**	**Maximum spacing**	**Physical range**		**Physical length**	**Recombination distance**	**Genome coverage**
		**cM**	**Marker cM^–1^**	**cM**	**bp**	**bp**	**bp**	**Kb/cM**	**%**
1	52	98.0	1.9	13.6	497,265	52,165,138	51,667,873	527.5	99.0%
2	111	102.1	0.9	6.0	195,928	49,033,712	48,837,784	478.4	99.6%
3	58	95.8	1.7	11.4	61,877	52,141,173	52,079,296	543.9	99.6%
4	74	80.6	1.1	5.6	90,666	45,793,192	45,702,526	567.0	99.4%
5	75	87.0	1.2	10.2	89,353	40,526,191	40,436,838	465.0	99.1%
6	48	61.6	1.3	6.8	521,607	31,379,071	30,857,464	501.1	96.5%
7	65	92.3	1.4	7.4	61,520	51,592,532	51,531,012	558.1	99.6%
8	82	96.4	1.2	9.5	159,002	59,613,870	59,454,868	616.9	99.7%
9	52	67.1	1.3	5.6	582,540	37,374,817	36,792,277	548.5	98.2%
10	44	57.4	1.3	9.3	190,638	43,194,406	43,003,768	749.7	99.4%
11	60	86.6	1.4	13.4	51,396	50,005,757	49,954,361	576.8	99.2%
Average	65.5	84.1	1.3	9.0				557.5	99.0%
Total	721	924.7					510,318,067		

#### Identification of Additive QTLs and Their Distribution on the Molecular Linkage Map

**FIGURE 5:**
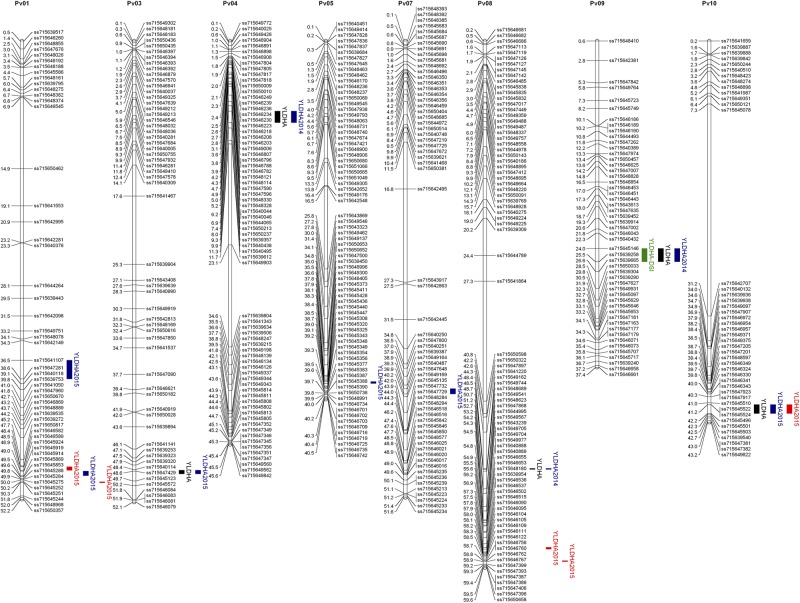
Joint chromosomal linkage map and QTLs for grain yield (YLDHA, kg ha^– 1^) and drought susceptibility index (DSI). The bars in blue represent the environments under full irrigation, red for terminal drought, black for the mean yield, and green for DSI. The position in the chromosome are the physical positions of the markers in Mbp and plotted with MapChart (Voorrips, 2002).

QTL analyses were performed for grain yield in each environment, consisting of 2 years grown in full irrigation, terminal drought in the second year, and the average yield across environments. The same analysis was conducted for the DSI calculated from the drought and well-watered conditions in 2015 (Table 4 and Figure 5). Significance thresholds for LOD scores, calculated by permutations for each environment, were 3.7 (environment 2014), 3.6 (2015c), and 3.6 (2015d). LOD scores for averages across environments had a threshold of 4.7. The threshold LOD score for DSI was 12.1. Twenty QTLs had LOD scores about the respective thresholds. They were distributed on eight chromosomes. These included three QTLs in 2014, five in 2015c, six in 2015d, five for the average across environments, and one for DSI.

**TABLE 4:** Summary of the QTL analysis for yield (kg ha^–1^) and drought susceptibility index (DSI) evaluated in three environments (2014 and 2015 under full irrigation, and 2015 under terminal drought).

**Chromo-some**	**Position (cM)**	**Year^1^**	**Treatment**	**LOD score**	**PVE^2^**	**Additive effect (kg ha^–1^) relative to SEA 5**			**Confidence interval**				**Potentially overlapping QTLs^3^**
						**PI 319441**	**PI 417653**	**PI 343950**	**Marker**		**Position**	**(Mbp)**	
1	45	2015	Well-watered	3.8	1.8	–8.3	–135.5	–36.5	ss715641107	ss715647281	36.46	38.63	
1	72	2015	Drought	4.6	1.0	99.4	0.0	111.0	ss715646589	ss715645924	48.52	48.90	Trapp et al. (2016)
1	76	2015	Well-watered	5.7	4.6	54.3	190.3	–59.7	ss715645914	ss715645869	48.99	49.48	
1	81	2015	Drought	6.0	4.8	–47.0	277.4	–7.4	ss715645252	ss715645251	50.22	50.30	
3	72	Mean		6.1	3.5	87.2	0.0	62.4	ss715639323	ss715639320	47.53	47.95	
3	72	2015	Well-watered	5.2	1.7	105.2	0.0	96.0	ss715639323	ss715639320	47.53	47.95	
4	33	Mean		4.8	1.9	–51.0	–44.5	–61.1	ss715650237	ss715639357	7.97	9.30	Blair et al. (2006)
4	33	2014	Well-watered	3.9	3.5	–85.1	–62.3	–56.6	ss715650237	ss715639357	7.97	9.30	
5	68	2015	Well-watered	4.5	1.3	–105.4	–60.4	–65.7	ss715645447	ss715645308	38.52	38.72	Trapp et al. (2016)
7	55	2015	Well-watered	8.0	2.6	–46.3	–12.7	–164.8	ss715640487	ss715647648	39.30	39.94	Hoyos-Villegas et al. (2016)
8	54	Mean		7.6	5.4	–75.4	–110.0	–73.5	ss715649162	ss715649744	48.44	48.51	
8	54	2014	Well-watered	4.6	4.7	–19.7	–96.7	–81.6	ss715649162	ss715649744	48.44	48.51	
8	78	2015	Drought	6.7	2.4	111.0	198.6	118.7	ss715646502	ss715646515	57.32	57.55	
8	87	2015	Drought	7.9	1.9	–33.2	115.2	–123.3	ss715646762	ss715646767	58.78	58.94	
9	33	2015	DSI	20.9	38.0	0.6	0.0	0.4	ss715645146	ss715639258	23.96	25.49	Mukeshimana et al. (2014), Berny Mier y Teran et al. (2019b)
9	33	Mean		6.1	3.2	–65.5	0.0	–80.1	ss715645146	ss715639258	23.96	25.49	
9	33	2014	Well-watered	4.1	4.1	–68.8	0.0	–90.9	ss715645146	ss715639258	23.96	25.49	
10	46	Mean		5.1	2.8	17.2	–52.5	–71.1	ss715645524	ss715645496	41.21	42.15	Hoyos-Villegas et al. (2016)
10	46	2015	Well-watered	3.6	3.2	116.4	–46.7	–102.0	ss715645524	ss715645496	41.21	42.15	
10	46	2015	Drought	3.8	0.6	10.0	–25.6	–100.5	ss715645524	ss715645496	41.21	42.15	

*^1^Mean: average of the three treatments. ^2^Percentage of variation explained. ^3^For further explanations, see the section “Discussion.”*

**FIGURE 6:**
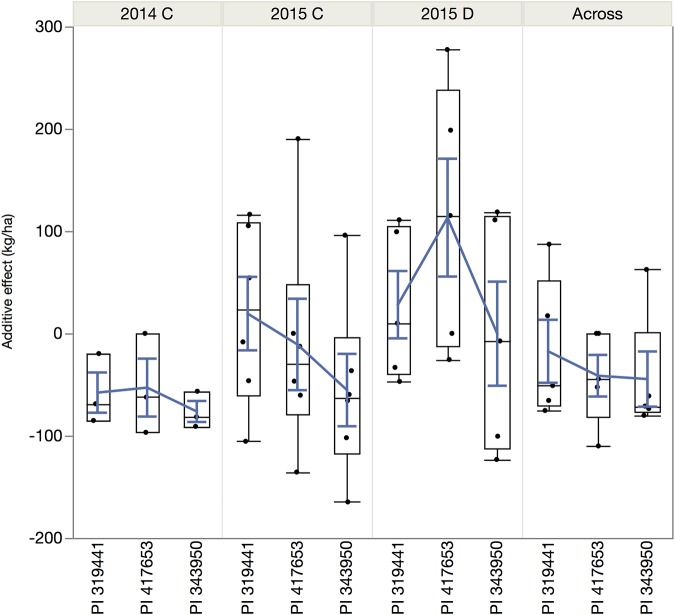
Quantile boxplot and overall mean (blue line) of the additive effect of the alleles of the wild accessions in comparison to the reference allele from SEA 5, the shared domesticated parent.

The magnitude of these QTLs ranged from 0.6% to 5.4% with an outlier at 38% (for DSI on chromosome 9, observed in the dw319441 and dw343950 populations). The allelic effects of the chromosome regions marked by these QTLs varied considerably among the three accessions between negative and positive values. The most negative value (−164.8 kg ha^–1^) was for yield QTL Pv07.55 in 2015, inherited from PI 343950. The largest positive allelic effect (277.4 kg ha^–1^) was observed in well-watered 2015 conditions and was inherited from PI 417653. Overall, the average allele effect of PI 343950 was the lowest in all environments and in the mean across environment (Figure 6 and Table 4). In contrast, the allele effect of PI 319441 was higher than that of PI 417653 in 2014 and 2015, both under full irrigation, while the average allele effect of PI 417653 was higher than that of PI 319441 under drought in 2015. In addition, the allele effects across genotypes were relatively higher under drought than under full irrigation in 2015. The average QTL significance interval was about 0.8 Mbp, ranging between 0.07 Mbp (on Pv08) and 2.18 Mbp (on Pv01). Many but not all QTL intervals appeared to be located toward the extremities of the chromosomes (Table 4).

## Discussion

### Population Development and Segregation Distortion

The main objective of the investigation was to survey eco-geographic adaptive variation in wild beans as a source of novel alleles to increase productivity, as well as to test if these alleles have differential expression under drought constraints. A resource commonly used for genetic studies are diversity panels, which allow a large sample of the variation to be tested and higher genetic resolution to be obtained. Prior to this study, a panel of wild accessions of the Mesoamerican gene pool was evaluated in a greenhouse setting revealing phenotypic variation in root and shoot traits, as well as genomic regions controlling these traits (Berny Mier y Teran et al., 2019a). However, these studies are limited by the confounding effects of population structure and relatedness, as well as low power of detection of rare alleles (Anderson et al., 2011; Bazakos et al., 2017). Population structure is even more geographically constrained in wild bean populations than in its domesticated forms, as dispersal and intercrossing between wild populations are limited compared with domesticated populations (Papa and Gepts, 2003; Zizumbo-Villarreal et al., 2005). In addition, some wild forms, including in common bean, are not adapted to cultivated conditions, due to their profuse and extended climbing growth habit and photoperiod sensitivity (Acosta-Gallegos et al., 2007).

Multiparent populations have several advantages over biparental populations for genetic studies: an increase in allelic diversity, sample size at a specific locus (increasing the power of detection), and mapping resolution (Garin et al., 2017; Holland, 2015). Various schemes for multiparent populations have been proposed; they are mostly divided into two groups: intermating of many parental lines and interconnected biparental populations (Fouilloux and Bannerot, 1988; Cavanagh et al., 2008; Buckler et al., 2009; Holland, 2015). Here, three populations were developed from domesticated by wild crosses using an elite domesticated breeding parent, selected for drought tolerance, as the common parent and three wild accessions from a range of rainfall conditions from the driest part of the distribution in Durango, northern Mexico, to a high-rainfall region in highland Guatemala. A domesticated genotype as the common parent allows for comparison of QTLs contributed by the different wild genotype relative to each other and to those of the domesticated parent. Domesticated × wild nested populations have been developed in maize (Liu et al., 2016) and barley (Schnaithmann et al., 2014; Maurer et al., 2015; Nice et al., 2016). In all cases, the populations were developed after one to four backcrosses to the domesticated parent.

Inbred-backcrossed lines facilitate QTL detection (Kaeppler, 1997) as the lines are more homogeneous and their interaction with other traits can be defined more precisely. For example, the benefit of earliness as an escape for terminal drought can be better assessed in this type of population after measuring earliness and yield under different irrigation regimes (Beebe et al., 2014; Polania et al., 2017). In addition, if superior lines are identified in domesticated × wild inbred backcross populations, few or no additional backcrosses are needed for breeding use (Tanksley and Nelson, 1996). However, if a trait is controlled epistatically by one or more loci without independent additive effect in the wild, it will be more difficult to identify such epistatic interactions in backcross populations (Tanksley and Nelson, 1996; Kaeppler, 1997; Johnson and Gepts, 2002). Only one backcross generation was used to limit the loss of detection power of the effect of wild alleles as every added backcross decreases the number of alleles from the wild parent and, hence, the power of QTL detection (Kaeppler, 1997).

Nested populations have been developed and used to study an array of traits in maize (Buckler et al., 2009; Wu et al., 2016; Xiao et al., 2016), soybean (Song et al., 2017), rice (Fragoso et al., 2017), barley (Saade et al., 2016), wheat (Bajgain et al., 2016), and common bean (Hoyos-Villegas et al., 2016), among others. While wild by domesticated nested populations exist in maize (Liu et al., 2016) and barley (Schnaithmann et al., 2014; Maurer et al., 2015; Nice et al., 2017), ours is the first domesticated by wild nested population in common bean. Besides the beneficial alleles for yield, these three populations could be of great use as breeding material and for future evaluations as the domesticated parent and the wild accessions can be polymorphic for other potentially useful traits. In evaluations of wild germplasm, PI 319441 was found to have high sulfur amino acid content in the seed and a high degree of protein hydrolysis after cooking (Montoya et al., 2008), a high content in polyphenols (Espinosa-Alonso et al., 2006), a high iron concentration (Blair et al., 2013) and, thus, could be used as a source for improved nutrition. PI 417653 had a high root efficiency ratio (total P content: root area) in low nitrogen conditions (Araújo et al., 1998), a high level of resistance to cucumber mosaic virus (Griffiths, 2002), and medium tolerance to salinity during early vegetative growth (Bayuelo-Jiménez et al., 2002).

The average wild allele frequency in our populations was 0.23, very close to the expected frequency of 0.25 in a biparental population with a single backcross. Allele frequencies significantly different from the expected frequency can be due to genetic mechanisms of segregation distortion, selection against photoperiod sensitivity and late flowering, or unintended selection. Processes that lead to segregation distortion include gametic incompatibility, genetic load, and asymmetric allelic inheritance in heterozygotes (Bodénès et al., 2016; Lyttle, 1991) besides the selection applied to the populations during their development. Genomic regions of low wild allele frequency were found in various chromosomes. In Pv01, an area of low frequency in all three populations was found around the 48 Mb position. The low frequency of wild alleles is likely due to the presence of a photoperiod sensitivity gene, *Ppd*, identified in this region (Koinange et al., 1996; Kwak et al., 2008; Weller et al., 2019) and selected against during population development (Figure 1). Segregation distortion in this region has also been found in two biparental wild by domesticated common bean populations (Blair et al., 2006; Blair and Izquierdo, 2012).

At least two other photoperiod loci have been identified, but have not been mapped as yet: the locus *Hr*, which is recessive and hypostatic to *Ppd* (Gu et al., 1998; Kwak et al., 2008) and the locus *Tip*, which is also recessive and increases earliness at cooler temperatures in long daylength (White et al., 1996). *Tip* might be allelic to either *Ppd* or *Hr* (White et al., 1996). Other regions controlling quantitatively photoperiod sensitivity have been located on Pv03 and Pv04 (Wallach et al., 2018).

There were other regions almost devoid of wild alleles at the end of chromosome Pv02 in one population (dw417653) and at the end of Pv07 in two populations (dw319441 and dw417653). Distortion in the latter region was also observed by Blair et al. (2006). In contrast, some regions in Pv02 and Pv11 showed a wild allele frequency of 0.4, that is, higher than the expected wild allele frequency after a single backcross. Nevertheless, although QTL analyses assume low segregation distortion, including distorted markers does not necessarily increase false positives or bias the effect and position, especially in large populations (Xu, 2008).

### Field Evaluation

The three populations were evaluated under field conditions in 2 years under optimal irrigation conditions. In the second year, a terminal drought stress was imposed by withdrawing the final two irrigations. Thus, the three populations were tested in a total of three environments. The analysis of variance showed a significant effect of population, environment, and their interaction, for yield production. The environmental effect was larger than the population and interaction effects. The overall yield in 2014 was almost half that of 2015 and the correlation between treatments was higher between drought and full irrigation in 2015, than between full irrigation in 2014 and full irrigation in 2015 and drought in 2015. This might be explained by the effect of hot weather experienced during flowering in 2014. In addition, terminal drought resulted in a marked 17% yield reduction relative to the irrigated treatment. Nevertheless, the ranks of the populations within environments were similar. The dw417653 population was higher-yielding than the dw319441 populations, which, in turn, was higher-yielding than the dw343950 population.

The wild parent of the latter population originated in the warmest and wettest climate (23.5°C, 1600 mm) of the three wild parental lines, suggesting that variation for increased productivity can be found in drier and cooler areas. However, the wild parent of population dw417653, which had the highest yield, originated in a slightly warmer (19.4°C vs. 18°C) and wetter (733 mm vs. 588 mm) environment as the second-ranked population (PI 319441). Thus, there may not be a linear relationship between the aridity of the environment of origin and the ability to increase yields in domesticated × wild crosses. Other factors may play a role in addition to aridity adaptation, such genetic distance between the wild accession and the domesticated gene pool. PI 417653 (G12910) has been implicated in the Mesoamerican domestication of common bean (Kwak et al., 2009).

### Map Development and Joint Linkage QTL Analysis

A consensus map from the three populations was built, which was adequately dense for joint linkage analysis and subsequent QTL mapping (Table 3). Furthermore, the map covered 99% of the G19833 reference genome (Schmutz et al., 2014). Through joint linkage mapping, 20 QTLs for grain yield were identified in the individual environments (year and irrigation regime), the overall grain yield across environments, and the drought susceptibility index calculated from the drought effect in the second year. Among the 20 QTLs, there were 13 non-overlapping QTLs. Two QTLs on chromosome Pv01 at 76 and 81 cM were tightly linked genetically and physically and had similar additive effect patterns among wild alleles. It is possible, therefore, that they are the same QTL. On the same chromosome, a QTL was mapped at the 72 cM position; however, the pattern of additive effects was reversed from that at the 76 and 81 cM positions, suggesting a different QTL. On Pv07, QTLs at the 78 and 87 cM positions had different additive effect patterns for two of three parental loci suggesting these two QTLs are distinct.

From the 20 QTLs, three were detected in 2014, five in 2015 under full irrigation, six in 2015 under terminal drought, five in the overall average across environments and one for DSI. From these, four QTLs were unique to 2015C and three to 2015D. The variation explained by the QTLs ranged from 0.6 to 5.4%, expected for a highly polygenic trait such as yield (Johnson and Gepts, 2002; Blair et al., 2006). For all QTLs that were expressed in different environments, the additive effects were of similar sign, suggesting that there were no tradeoffs between years or treatments. Although for most QTLs the effect of the wild alleles showed the same sign, for some, e.g., Pv08.87, the allele of PI 417653 had a positive effect (115 kg ha^–1^) while the alleles of PI 343950 and PI 319441 had a negative effect (−123 kg ha^–1^ and −33 kg ha^–1^, respectively). This observation suggests that this QTL consists of an allelic series at one locus or represents several, linked loci (Buckler et al., 2009). The detection of allelic series is one of the advantages of nested populations compared with bi-allelic variation in genome-wide association of diversity panels or other multiparent populations (Brachi et al., 2011).

Furthermore, within and across environments, the alleles of PI 343950, the wild population from the wettest location, had the lowest average additive effect. Conversely, the average allelic effects of the accessions from the drier habitats were higher: PI 319441 alleles in both 2014 and 2015, both under full irrigation, whereas PI 417653 alleles were higher under drought in 2015. The overall allele effect of PI 319441 was higher than that of PI 417653 in 2014 and 2015, both under full irrigation, while the average allele effect of PI 417653 was higher than that of PI 319441 under drought in 2015. This suggest that drought tolerance could be a driver of local adaptation. Some traits, like deeper rooting, water use efficiency, earliness in flowering and maturity (Berny Mier y Teran et al., 2019a), might be also beneficial under cultivation if growing seasons are shorter. Berny Mier y Teran et al. (2019a) observed that PI 417653, the wild accession with the strongest allelic effects under drought, has deeper roots and the fastest early growth (Days to the V3 stage) compared to the other two wild accessions. On the contrary, high rainfall conditions could increase selection pressure toward more vigorous vegetative growth and higher disease resistance.

There are two previously published wild by domesticated yield QTL analyses. Blair et al. (2006) evaluated a cross between a domesticated Andean cultivar (ICA Cerinza) and a wild type from Guatemala (G24404). They found nine QTLs for grain yield, with four of them having the wild allele increasing the trait. A QTL on Pv04 overlapped with our findings, which was detected in 2014 and as average across the three environments (Table 4). Their confidence interval was 0.4 to 9.5 Mbp while the QTL identified in this study was located within a 7.9 to 9.3 Mbp interval (Table 4). However, the additive effect of the G24404 accession was positive while the alleles from the three wild sources in our study had a negative effect. It is possible that the allele from the Andean gene pool in ICA Cerinza had a relatively smaller effect than that of SEA 5 and the wild accessions. Blair and Izquierdo (2012) evaluated a population of the same domesticated cultivar (ICA Cerinza) crossed to G10022 (PI 319441), one of the parents of the nested populations studied here. They found one QTL on Pv05, with the domesticated allele having the positive effect. This QTL did not overlap with Pv05.68 of the current investigation. By comparing the current results with our previous evaluation of a panel of wild germplasm (Berny Mier y Teran et al., 2019a), the QTL in Pv10 located in the interval of 41.2 to 42.1 Mb was situated near a SNP at 38.3 Mb associated with total biomass in the wild panel. This QTL showed a positive additive effect on yield resulting from one parental allele (PI 417653) but a negative effect from the other two parents, suggesting that this genomic location might be involved in local adaptation within the wild germplasm.

Two domesticated by domesticated mapping populations have been developed using SEA 5 (Briñez et al., 2017; Mukeshimana et al., 2014). The latter found three QTLs for yield, with one QTL at Pv09 (SY9.1, confidence interval of 25.1–27.1 Mb) overlapping with our findings (Mukeshimana et al., 2014). The allele of SEA 5 had a negative effect compared to a parental line of the Andean gene pool, while SEA 5 had a positive effect when compared to the wild types in the present study. Briñez et al. (2017) developed a population based on SEA 5 crossed to an Andean accession. They found two QTLs for grain yield, which did not overlap with our findings. Within other published QTL analyses for yield, our findings overlapped with QTLs found by Trapp et al. (2016) in Pv01 (SY1.1) and Pv05 (SY5.1), by Hoyos-Villegas et al. (2016) in Pv07 (SY7.4) and Pv10 (SY10.1), and by Berny Mier y Teran et al. (2019b) in Pv09. The latter QTL also overlapped with the SY9.1 QTL found by Mukeshimana et al. (2014).

From the 13 non-overlapping QTLs in this study, five had at least one wild allele with a significant positive additive effect. The allele of PI 417653 at Pv01.81 had the largest effect (277 kg ha^–1^), detected under drought in 2015. PI 417653 was the only parent that had effects close to zero, in three of the 13 QTLs. This wild parent is part of the putative Mesoamerican domestication center of common bean in west-central Mexico and is, therefore, the closest genetically to the domesticated parent of the three wild populations used in this study (Kwak et al., 2009). This observation suggests that the ancestor of this wild population contributed yield alleles to the Mesoamerican domesticated gene pool or that it could harbor an introgression from the domesticated gene pool (Papa and Gepts, 2003; Papa et al., 2005).

### Putative Candidate Genes for QTLs

The QTLs detected in this study encompass genomic regions with high number of genes, in general. For example, the statistical significance region of the QTL at Pv09.33, which was detected in the 2014 well-watered treatment and for DSI, ranges from 24.43 to 25.98 Mb in version 2.1 of the common bean reference genome deposited in Phytozome^3^. Within this region, 105 distinct gene models have been identified. Their annotation (obtained from PFAM^4^ and PANTHER^5^; see Supplementary Table S1) reveals genes implicated in a variety of cellular processes and molecular functions, such as response to stress, oxidative response, signal transduction, protein ubiquitination, chromosome modification, histone modification, metal ion binding, and RNA processing. For example, within this QTL region, Phvul.009G164600.1 was annotated as a serine carboxypeptidase, which was described as involved in oxidative stress in rice (Liu et al., 2008). Phvul.009G169400.2 is related to callose synthase, involved in callose deposition, a functional category that was also described by Recchia et al. (2018), in an experiment with BAT477 under drought and well-watered conditions, in the presence or absence of arbuscular mycorrhizal fungi. Moreover, this QTL region also contained members of a leucin-rich repeat family. This group of genes belongs a larger protein family of receptor-like kinases, playing important roles in stress resistance (Ye et al., 2017), a category also found by Recchia et al. (2018) in common bean. However, because the genomic significance region of a low-heritability trait like yield is so large, an exhaustive list of gene models for each QTL identified in this study becomes quite long as illustrated in Supplementary Table S1. It is difficult to focus on likely candidate gene models without further experimentation, which falls beyond the scope of this work.

An alternative approach is to map genes with a putative role in drought-tolerance and examine to what extent they co-segregate with QTLs. Genome-wide categorizations have been published for a few families in common bean, such as aquaporins (AQP) (Ariani and Gepts, 2015) and Dehydration Responsive Element-Binding (DREB) genes (Cortés et al., 2012; Konzen et al., 2019). AQPs play important roles as water channel proteins in plants. The AQP gene PvPIP1;1 is located near a QTL detected under drought in 2015 on chromosome Pv01, with a distance of approximately 120 Kbp from marker ss715645251. Two transcription factors belonging to the DREB gene family, traditionally characterized as involved in abiotic stress responses such as drought, were located within a specific QTL. Phvul.001G136100 is located within the QTL at Pv01.45, a QTL detected in the 2015 well-watered treatment and Phvul.003G241700 is located at Pv03.72, a QTL detected in the well-watered treatment in 2015. Both genes were previously categorized as DREB2 genes, which are normally involved in responses to abiotic stresses such as caused by water deficit (Konzen et al., 2019).

## Conclusion

Our original hypothesis was that wild *P. vulgaris* populations from drier areas would be better sources of yield-enhancing genes in a domesticated line than those from more humid areas. We showed here that this is indeed the case. However, we also showed that these same wild populations from drier areas also increased yields under well-watered conditions. On average, the superior progeny lines from arid wild beans increased yield by around 20%. Taken together, various wild genomic regions were identified that had positive effects on yield under well-watered and drought-stress conditions. Our results have the potential to make future introgressions assisted with markers faster and more efficient. The alleles with positive (and negative) effects help explain the transgressive segregation found in this study and underscore the potential of wild variation to improve the productivity of domesticated beans. Future work is needed to validate the QTLs with a positive effect on yield introgressed in different domesticated genetic (Des Marais et al., 2013; Shen et al., 2018). Variation within some QTLs in the magnitude of the effect or sign of the effect suggest allelic series associated with a range of phenotypic variation. This variation could be the basis to local adaptation (Buckler et al., 2009; Kronholm et al., 2012). In addition, further sampling of the wild variation is warranted (Stich, 2009), as well as evaluation in wetter and more humid conditions than in the field site in California.

## Data Availability Statement

The datasets generated for this study can be found in Dryad https://doi.org/10.25338/B8FW3M.

## Author Contributions

JB and PG designed the experiments. JB, AP, and EK carried out the field trial and genotyping. JB analyzed the data and drafted the manuscript. JB, EK, AP, ST, and PG contributed to and edited the manuscript.

## Conflict of Interest

The authors declare that the research was conducted in the absence of any commercial or financial relationships that could be construed as a potential conflict of interest.

## Footnotes

1 www.worldclim.org

2 https://soilseries.sc.egov.usda.gov

3 https://phytozome.jgi.doe.gov/pz/portal.html

4 https://pfam.xfam.org/

5 http://pantherdb.org/
